# Acute beetroot juice consumption does not alter cerebral autoregulation or cardiovagal baroreflex sensitivity during lower-body negative pressure in healthy adults

**DOI:** 10.3389/fnhum.2023.1115355

**Published:** 2023-01-19

**Authors:** Morgan L. Worley, Emma L. Reed, Christopher L. Chapman, Paul Kueck, Lauren Seymour, Triniti Fitts, Hannah Zazulak, Zachary J. Schlader, Blair D. Johnson

**Affiliations:** ^1^Department of Exercise and Nutrition Sciences, School of Public Health and Health Professions, University at Buffalo, Buffalo, NY, United States; ^2^Department of Human Physiology, Bowerman Sports Science Center, University of Oregon, Eugene, OR, United States; ^3^Department of Kinesiology, School of Public Health, Indiana University Bloomington, Bloomington, IN, United States

**Keywords:** cerebrovascular function, dietary nitrates, cerebral blood flow, lower-body negative pressure, blood pressure control

## Abstract

**Introduction:**

Beetroot juice (BRJ) improves peripheral endothelial function and vascular compliance, likely due to increased nitric oxide bioavailability. It is unknown if BRJ alters cerebrovascular function and cardiovagal baroreflex control in healthy individuals.

**Purpose:**

We tested the hypotheses that BRJ consumption improves cerebral autoregulation (CA) and cardiovagal baroreflex sensitivity (cBRS) during lower-body negative pressure (LBNP).

**Methods:**

Thirteen healthy adults (age: 26 ± 4 years; 5 women) performed oscillatory (O-LBNP) and static LBNP (S-LBNP) before (PRE) and 3 h after consuming 500 mL of BRJ (POST). Participants inhaled 3% CO_2_ (21% O_2_, 76% N_2_) during a 5 min baseline and throughout LBNP to attenuate reductions in end-tidal CO_2_ tension (PETCO_2_). O-LBNP was conducted at ∼0.02 Hz for six cycles (−70 mmHg), followed by a 3-min recovery before S-LBNP (−40 mmHg) for 7 min. Beat-to-beat middle cerebral artery blood velocity (MCAv) (transcranial Doppler) and blood pressure were continuously recorded. CA was assessed using transfer function analysis to calculate coherence, gain, and phase in the very-low-frequency (VLF; 0.020–0.070 Hz) and low-frequency bands (LF; 0.07–0.20 Hz). cBRS was calculated using the sequence method. Comparisons between POST vs. PRE are reported as mean ± SD.

**Results:**

During O-LBNP, coherence_*VLF*_ was greater at POST (0.55 ± 0.06 vs. 0.46 ± 0.08; *P* < 0.01), but phase_*VLF*_ (*P* = 0.17) and gain_*VLF*_ (*P* = 0.69) were not different. Coherence_*LF*_ and phase_*LF*_ were not different, but gain_*LF*_ was lower at POST (1.03 ± 0.20 vs. 1.12 ± 0.30 cm/s/mmHg; *P* = 0.05). During S-LBNP, CA was not different in the VLF or LF bands (all *P* > 0.10). Up-cBRS and Down-cBRS were not different during both LBNP protocols.

**Conclusion:**

These preliminary data indicate that CA and cBRS during LBNP in healthy, young adults is largely unaffected by an acute bolus of BRJ.

## Introduction

Nitric oxide is an important modulator of vascular tone, blood flow, and tissue oxygenation (Levine et al., 2012). The production of nitric oxide can be catalyzed by three isoenzymes of nitric oxide synthase (NOS): endothelial NOS, neuronal NOS, and inducible NOS. Endothelium NOS and neuronal NOS catalyze the production of nitric oxide from L-arginine (Llorens et al., 2002). Once nitric oxide is produced in the endothelium, it is then freely able to diffuse from endothelial cells into the vascular lumen or into vascular smooth muscle cells where soluble guanylyl cyclase is activated to form cyclic guanosine monophosphate (Ahmad et al., 2018). This pathway promotes vasodilation and has important implications for vascular health (e.g., control of cerebral blood flow) (Shu et al., 2015). Neuronal NOS, which also catalyzes the production of nitric oxide from L-arginine, is expressed in central and peripheral neurons. Thus, production of nitric oxide *via* neuronal NOS has important implications for cognitive function (e.g., memory) (Ally et al., 2020). Reductions in endogenous nitric oxide occur with aging have been identified as a risk factor for disease states such as cardiovascular disease (Naseem, 2005) and Alzheimer’s disease (Venturelli et al., 2018). Cardiovascular disease and Alzheimer’s disease are associated with impaired vascular function, including endothelial dysfunction (Widlansky et al., 2003; Lidder and Webb, 2013), increased arterial stiffness (Hughes et al., 2015, 2018; Bonarjee, 2018), and reduced cerebral blood flow (Mazza et al., 2011; Roy et al., 2017; Korte et al., 2020).

Endogenous nitric oxide production can be supplemented with dietary nitrates to increase its bioavailability (Capper et al., 2022). Dietary nitrate supplementation has been shown to lower blood pressure (Houston and Hays, 2014), and improve endothelial function (Houston and Hays, 2014; Sindler et al., 2014; Walker et al., 2019; Rossman et al., 2021) and vascular compliance (Houston and Hays, 2014), in adults who are normotensive or hypertensive (Lidder and Webb, 2013). These improvements reflect beneficial changes in the peripheral vasculature, but it is less clear if nitrates alter mechanisms of cerebrovascular control. A single dose of the nitric oxide donor, nitroglycerin, reduces cardiovagal baroreflex sensitivity (cBRS) and increases sympathetic baroreflex sensitivity (Hamaoka et al., 2021). Thus, it is biologically plausible that acute increases in nitric oxide bioavailability *via* dietary nitrate supplementation alter blood pressure control, and in turn alter the regulation of cerebral blood flow due to integrative mechanisms.

Beetroot juice (BRJ) is a popular nitric oxide supplement due to its high inorganic nitrate profile. When BRJ is ingested, nitrate is broken down to nitrite in the oral cavity *via* anaerobic bacteria and is further reduced to nitric oxide in the stomach before entering the bloodstream (Ma et al., 2018). Acute BRJ consumption (150 mL) augments plasma nitrate and nitrite concentrations, which reduces blood pressure and improves cognition at 2.5 and 3.25 h post-ingestion in younger and older adults (Stanaway et al., 2019). Improved cognition might be associated with acute increases in regional cerebral perfusion (Presley et al., 2011), but the effects on cerebrovascular function remain unclear. Cerebral autoregulation (CA) is an important marker of cerebrovascular function because it protects the brain against hypo- and hyper-perfusion during large fluctuations in blood pressure. These fluctuations in blood pressure may occur spontaneously throughout the day but are more pronounced in response to a stimulus [e.g., postural changes, lower-body negative pressure (LBNP)]. During fluctuations in blood pressure, arterial baroreceptors reflexively alter neural signaling to modify heart rate and systemic vascular resistance to control arterial blood pressure (Swenne, 2013). Simultaneously, pial arteries and cerebral arterioles simultaneously alter vascular resistance to modulate cerebral blood flow (Sekiguchi et al., 2014). Maintaining arterial blood pressure and cerebral blood flow requires the integrated control of cBRS and CA.

The purpose of the present study was to determine if acute BRJ consumption alters cBRS and CA. We tested the hypotheses that a single bolus of BRJ would acutely increase dynamic and static CA and reduce cardiovagal-baroreflex sensitivity during oscillatory and static LBNP in healthy, young adults. These LBNP protocols are experimental models used to examine the dynamic and static components of cerebral blood flow regulation and autonomic control of blood pressure.

## Materials and methods

### Participants

This study includes data from participants that were tested in separate studies that investigated the effects of mild hypercapnia and acute BRJ consumption on renal hemodynamics (Chapman et al., 2020, 2021). These studies utilized similar measurements and protocols, but tested separate novel hypotheses. In the present study, thirteen healthy adults (age: 26 ± 4 years; height: 171 ± 9 cm; weight: 69 ± 11 kg; body mass index: 23 ± 3 kg/m^2^; 5 women) completed the study. All participants were fully informed of the experimental procedures prior to providing written, informed consent. Participants were excluded from the study if they self-reported to use tobacco or medications known to affect blood pressure or the autonomic nervous system (e.g., beta-blockers, angiotensin converting enzyme inhibitors); had a pre-existing autonomic, cardiovascular, metabolic, respiratory, or endocrine disorder; major depression; were pregnant or breastfeeding. Women were tested during the first 10 days of their self-identified menstruation (i.e., onset of menses) to control for the menstrual cycle (Favre and Serrador, 2019). The study was approved by the Institutional Review Board at the University at Buffalo and was performed in accordance with the standards set by the latest revision of the Declaration of Helsinki except for registration in a database.

### Experimental approach

Participants completed one study visit that consisted of oscillatory lower-body negative pressure (Oscillatory-LBNP) and static lower body negative pressure (Static-LBNP) before and 3 h after BRJ consumption to allow for plasma nitrite levels to peak (Webb et al., 2008; Casey et al., 2015). Participants were instructed to abstain from medications, alcohol, exercise, and caffeine for 12 h, and food for 2 h prior to reporting to the laboratory. Participants were also instructed to abstain from antibacterial mouthwash and chewing gum the morning of the study as this can hinder the breakdown of dietary nitrates (Govoni et al., 2008). Upon arrival, a urine sample was obtained to ensure euhydration (urine specific gravity <1.020) and negative pregnancy status (women only). Women also reported the start date of their last menstrual cycle.

Participants laid in the supine position for the duration of instrumentation and measurements. A neoprene skirt was used to seal the participants’ lower body at the level of the iliac crest into an airtight chamber for LBNP testing (described below). Participants were then instrumented with physiological monitoring equipment. Then, after >10 min of supine rest, 5 min of baseline data were recorded before initiating the LBNP protocols. To attenuate reductions in PETCO_2_ that can be observed during LBNP, participants inhaled a mild hypercapnic gas (FiCO_2_: 0.03, FiO_2_: 0.21, FiN_2_: 0.76) during baseline and throughout the duration of testing. Our lab has previously demonstrated that LBNP set to −40 mmHg for 5 min reduces PETCO_2_ by 5 ± 3 mmHg in healthy, young adults (Worley et al., 2021), which likely influences cerebrovascular responses to LBNP. However, elevated FiCO_2_ can also impair CA. This has been reported using an FiCO_2_ of ≥0.05 (Panerai et al., 1999; Perry et al., 2014), thus we selected an FiCO_2_ of 0.03 to attenuate potential reductions in PETCO_2_ that may occur during LBNP, thereby reducing the risk of altering CA.

Following the 5-min baseline, the Oscillatory-LBNP protocol commenced. Oscillatory-LBNP was conducted at a frequency of ∼0.02 Hz, which consisted of 30 s on (−70 mmHg) and 30 s off (0 mmHg) for a total of six cycles. Then, a 3-min recovery period was allotted before the Static-LBNP protocol commenced. Static-LBNP consisted of −40 mmHg for a total of 7 min to assess static CA (Claassen et al., 2016; Panerai et al., 2022). Upon completion of Static-LBNP, participants were de-instrumented and consumed 500 mL of commercially available BRJ (listed as ∼1,500 mg/L of nitrate) (Biotta, Carmel, IN, USA) (Kenjale et al., 2011; Bond et al., 2013) within 10 min. The average concentration of nitrates measured in Biotta BRJ has been reported previously as 18,708 μmol/L, respectively (Casey et al., 2015). The testing protocol was then repeated 3 h after BRJ consumption. During this 3 h period, participants rested quietly in the laboratory (e.g., watched a non-stimulating video) and were allowed to consume 250 mL of water during the break.

The LBNP protocols had pre-defined termination endpoints, including (a) Sustained systolic blood pressure (SBP) <80 mmHg with a precipitous decrease in heart rate; (b) If participants self-reported any symptoms of pre-syncope (e.g., sweating, tunnel vision, nausea, dizziness); or (c) Upon participant request (Rickards et al., 2015). All participants completed the Oscillatory-LBNP protocol. One male participant did not complete the entire 7 min Static-LBNP protocol at pre-BRJ. This test was terminated at 6 min due to relative bradycardia with reduced SBP.

### Instrumentation and measurements

Height was measured with a stadiometer (Sartorius, Bohemia, NY, USA). Nude body weight was measured on an electronic scale to the nearest 0.01 kg in a private bathroom. Urine specific gravity was measured *via* a refractometer (Atago USA, Bellevue, WA, USA). Heart rate was continuously measured using 3-lead electrocardiogram (DA100C, Biopac Systems, Goleta, CA, USA). Beat-to-beat blood pressure was non-invasively measured using finger photoplethysmography on the left hand (middle finger) at heart level. Finger blood pressure was calibrated to brachial artery blood pressure in the supine position using the return-to-flow module (Finometer Pro, FMS, Amsterdam, Netherlands). Blood pressure was confirmed intermittently throughout the protocol using auscultation of the right brachial artery (electrosphygmomanometry; Tango M2; SunTech, Raleigh, NC, USA). End-tidal CO_2_ tension (PETCO_2_) was continuously measured to estimate arterial CO_2_, from a mouthpiece using capnography while the nose was occluded with a nose clip (Nonin Medical, Inc., Plymouth, MN, USA). Left middle cerebral artery blood velocity (MCAv) was continuously measured using a 2 MHz probe (DWL USA, Inc., Germany, Europe) with insonation techniques outlined by Willie et al. (2012). The anatomical location for the left MCA was marked by indelible ink so that the transducer placement for pre- and post-BRJ testing was the same. Additionally, the settings for depth and gain were kept the same for pre- and post-BRJ testing.

### Data analysis

Heart rate (1000 Hz), blood pressure (62.5 Hz), PETCO_2_ (62.5 Hz), and MCAv (1000 Hz) were continuously recorded using a data acquisition system (Biopac MP150, AcqKnowledge 4.2.0, Goleta, CA, USA). Stroke volume was estimated using Modelflow (Wesseling et al., 1985). Cardiac output was calculated as the product of heart rate and stroke volume. Cerebrovascular conductance index (CVCi) was calculated as the quotient of MCAv and mean arterial pressure. Mean values were extracted for all variables during the last minute of supine rest preceding Oscillatory-LBNP (baseline Oscillatory-LBNP) and Static-LBNP (baseline Static-LBNP), every interval of Oscillatory-LBNP, and every minute of Static-LBNP. Data are presented as the mean with 95% confidence intervals.

### Cerebral autoregulation analysis

**FIGURE 1:**
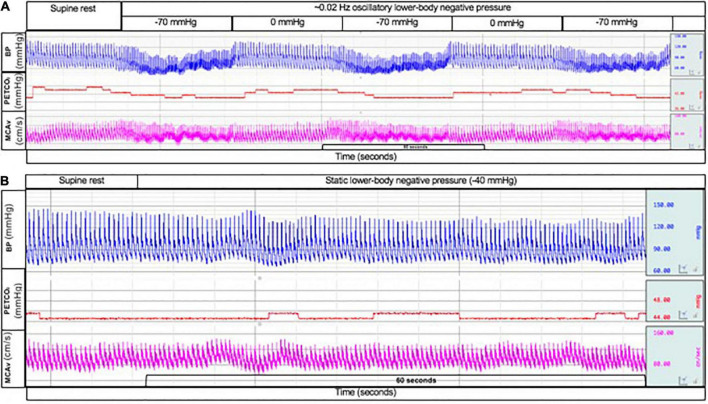
Typical tracing of beat-to-beat blood pressure (top; blue line), end-tidal CO_2_ tension (middle; red line), and middle cerebral artery blood velocity (bottom; pink line) during oscillatory lower-body negative pressure at –70 mmHg and 0 mmHg **(A)** and during static lower-body negative pressure at –40 mmHg **(B)**.

Dynamic CA was assessed using Oscillatory-LBNP intervals of 30 s on (−70 mmHg) and 30 s off (0 mmHg) (∼0.02 Hz). This interval time was selected due to the cerebral vasculature’s ability to dampen changes in arterial pressure that occur slower than ∼30 s (0.03 Hz) (Brothers et al., 2009; Hamner et al., 2019). Static CA was assessed by applying static-LBNP (−40 mmHg) for 7 min (Claassen et al., 2016; Panerai et al., 2022). Raw data files of beat-to-beat blood pressure (input signal) and MCAv (output signal) were used to assess dynamic and static CA *via* transfer function analysis (WinCPRS, Absolute Aliens Oy, Turku, Finland). Please refer to the examples of typical tracings for oscillatory-LBNP and Static-LBNP protocols in Figure 1. These analog signals were integrated with the R-R intervals (RRIs) of the ECG and re-sampled at 5 Hz to align the beat-to-beat time series. Data were analyzed by fast Fourier transformation using a Hanning window with sliding bandwidth of 180 s and 50% overlap to reduce cross-spectral leakage. Using Welch’s method, coherence, phase, and gain were calculated across the 0.00−0.50 Hz frequency band (Panerai et al., 2022). Based on transfer function analysis recommendations, we calculated indices of CA in the standard bands defined as very-low-frequency (VLF; 0.02–0.07 Hz), low-frequency (LF; 0.07–0.20 Hz), and high-frequency (HF; 0.20–0.50 Hz) (Panerai et al., 2022) for both LBNP protocols. A critical coherence value was not implemented for the inclusion/exclusion of gain and phase estimates for the following reasons: (1) our oscillatory-LBNP was conducted in the VLF band (∼0.02 Hz), which can result in low coherence values due to blood pressure and MCAv exhibiting a non-linear relationship (Claassen et al., 2016; Panerai et al., 2022), and (2) low signal-to-noise ratio due to poor data quality was unlikely due to visual inspection and exclusion of data that had excessive artifact (see below). Additionally, within the VLF band, gain and phase estimates are not different when comparing low vs. high coherence data (Claassen et al., 2009). Transfer gain and phase reflect the magnitude and time delay between alterations in blood pressure and MCAv within a given frequency, respectively. A decrease in gain and increase in phase are indicative of an “improvement” in CA as this reflects a greater ability of the brain to buffer changes in arterial pressure. Negative phase values were excluded from averaging in frequency bands <0.10 Hz due to the “wrap-around” effect (Claassen et al., 2016). Additionally, raw data files of beat-to-beat blood pressure and MCAv were visually inspected for artifact. Participants were excluded from transfer function analysis if excessive artifact persisted beyond 3 beats [Oscillatory-LBNP: *n* = 1 (male); Static-LBNP; *n* = 3 (1 male)] (Claassen et al., 2016). One additional participant (male) was excluded from Static-LBNP analysis since pre-testing was terminated early due to pre-syncopal symptoms.

### Cardiovagal baroreflex sensitivity analysis

Raw data files of ECG and beat-to-beat blood pressure were used for offline analysis of cBRS (WinCPRS, Absolute Aliens Oy, Turku, Finland) during Oscillatory-LBNP (∼6 min) and Static-LBNP (7 min). The ECG and beat-to-beat blood pressure waveforms were visually inspected for artifact and ectopic beats (none were identified). cBRS was calculated using the sequence method (Bertinieri et al., 1985; Laude et al., 2009). Four successive increases or decreases in SBP (± 1 mmHg) that corresponded with appropriate changes in RRI (± 5 ms) were identified (Parati and Mancia, 2000). Sequences were deemed valid if the *R*^2^ value calculated from individual linear regression analyses between SBP and RRI was ≥0.85 (Parati and Mancia, 2000). The time delay between SBP and RRI was set to 1 beat (Gross et al., 2002). Sequences were determined separately for Up-BRS (i.e., concurrent increases in SBP and RRI) and Down-BRS (i.e., concurrent decreases in SBP and RRI).

### Statistical analysis

Data were analyzed using PRISM software (version 8, GraphPad Software, La Jolla, CA, USA). We used linear mixed-effects models to compare absolute mean values during Oscillatory-LBNP at PRE-BRJ and POST-BRJ. Linear mixed-effects models were also used to compare absolute mean values during Static-LBNP at PRE-BRJ and POST-BRJ. No statistical comparisons were made between LBNP protocols. If a linear mixed-effects model revealed a significant interaction or main effect (Tybout AaS, 2001), we used Bonferroni’s *post hoc* analyses to determine where differences occurred. Within LBNP protocols, we used two-tailed paired *t*-tests to determine if indices of dynamic CA and cBRS differed between PRE-BRJ and POST-BRJ. Lastly, Cohen’s d effect sizes were calculated for CA and cBRS between PRE-BRJ and POST-BRJ within each LBNP protocol (Lenhard and Lenhard, 2016). Cohen’s d effect sizes were interpreted as none (*d* = 0.0–0.1), small (*d* = 0.2–0.4), intermediate (*d* = 0.5–0.7), and large (*d* = 0.8 and above) (Cohen, 1988; Lakens, 2013). Statistical significance was set *a priori* at *P* ≤ 0.05. Data are presented as mean with 95% confidence intervals. Individual *p*-values are reported where possible.

## Results

### Oscillatory lower-body negative pressure

#### Hemodynamic response to oscillatory LBNP

**FIGURE 2:**
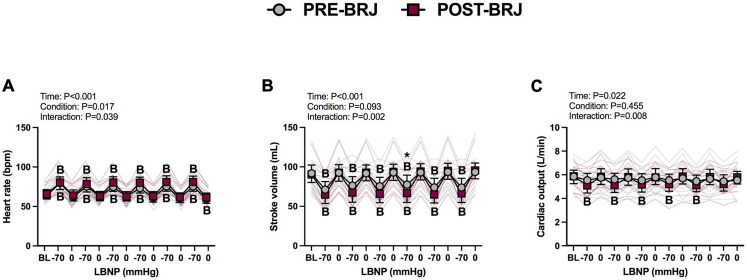
Individual responses and absolute means with 95% confidence intervals for heart rate **(A)**, stroke volume **(B)**, and cardiac output **(C)**, at the last minute of baseline (BL) and during each 30 s interval of oscillatory lower-body negative pressure (LBNP) before (PRE–BRJ) and 3 h after beetroot juice consumption (POST–BRJ). Linear mixed-effect models were used to compare mean values within a given variable. *Post-hoc* tests using the Bonferroni’s correction for multiple comparisons were used where appropriate. B = different from baseline (*P* ≤ 0.05). *Different from PRE-BRJ (*P* ≤ 0.05).

**FIGURE 3:**
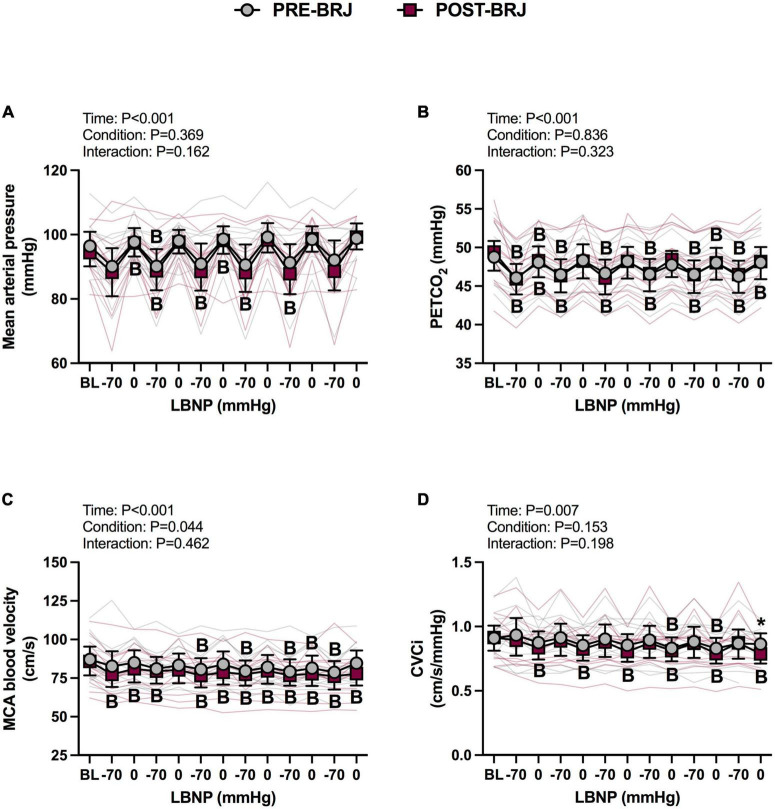
Individual responses and absolute means with 95% confidence intervals for mean arterial pressure **(A)**, end-tidal CO_2_ tension **(B)**, middle cerebral artery blood velocity **(C)**, and cerebrovascular conductance index **(D)** at the last minute of baseline (BL) and during each 30 s interval of oscillatory lower-body negative pressure (LBNP) before (PRE–BRJ) and 3 h after beetroot juice consumption (POST–BRJ). Linear mixed-effect models were used to compare mean values within a given variable. *Post-hoc* tests using the Bonferroni’s correction for multiple comparisons were used where appropriate. B = different from baseline (*P* ≤ 0.05). *Different from PRE-BRJ (*P* ≤ 0.05).

There were no differences between conditions at Baseline. During Oscillatory-LBNP, there was a significant condition effect for heart rate (*P* = 0.017), but multiple comparisons did not reveal significant differences (Figure 2A). A significant interaction effect was observed for stroke volume (*P* = 0.002), with multiple comparisons identifying that stroke volume was lower POST-BRJ at interval 4 of −70 mmHg in (*P* = 0.042) (Figure 2B). Despite an interaction (*P* = 0.008), cardiac output was not different between PRE-BRJ and POST-BRJ during Oscillatory-LBNP (Figure 2C). Mean arterial pressure, PETCO_2_, and MCAv were not different between PRE-BRJ and POST-BRJ during Oscillatory-LBNP (Figures 3A–C). CVCi was lower in POST-BRJ vs. PRE-BRJ at interval 6 of 0 mmHg (*P* = 0.023) (Figure 3D).

#### Dynamic cerebral autoregulation

Indices of dynamic CA during Oscillatory-LBNP are presented in Table 1. Coherence was greater at POST-BRJ in the VLF band (0.02–0.07 Hz) vs. PRE-BRJ (*P* = 0.008, *d* = 1.268). However, coherence was not different between PRE-BRJ and POST-BRJ in the LF (*P* = 0.510) or HF band (*P* = 0.137). Phase was not different between PRE-BRJ and POST-BRJ in any frequency band (all *P* ≥ 0.172). Gain was lower at POST-BRJ in the LF band (0.07–0.20 Hz) vs. PRE-BRJ (*P* = 0.049). Gain was not different between PRE-BRJ and POST-BRJ in the VLF (*P* = 0.669) or HF band (*P* = 0.352).

**TABLE 1:** Blood pressure, cerebral artery blood velocity, and indices of cerebral autoregulation indices of dynamic cerebral autoregulation during oscillatory lower-body negative pressure before and 3 h after beetroot juice (BRJ) consumption.

Variable	PRE-BRJ	POST-BRJ	*P*-value	Cohen’s d
MAP (mmHg)	94 (89, 99)	92 (87, 97)	0.306	0.220
MCAv (cm/s)	78 (71, 85)	75 (67, 82)	0.072	0.313
**Coherence (a.u.)**				
0.00–0.50 Hz	0.68 (0.63,0.73)	0.73 (0.68, 0.78)	0.097	0.591
0.02–0.07 Hz	0.46 (0.40, 0.51)	0.55 (0.51, 0.59)	0.008*	1.268
0.07–0.20 Hz	0.76 (0.68, 0.83)	0.78 (0.73, 0.83)	0.510	0.213
0.20–0.50 Hz	0.69 (0.62, 0.76)	0.74 (0.68, 0.81)	0.137	0.534
**Phase (degrees)**				
0.00–0.50 Hz	5 (1, 8)	5 (1, 8)	0.757	0.078
0.02–0.07 Hz	37 (22, 51)	43 (30, 55)	0.172	0.302
0.07–0.20 Hz	26 (21, 31)	24 (19, 29)	0.432	0.274
0.20–0.50 Hz	−7 (−14, 0)	−5 (−8, −2)	0.420	0.243
**Gain (cm/s/mmHg)**				
0.00–0.50 Hz	1.03 (0.86, 1.20)	0.98 (0.85, 1.11)	0.095	0.230
0.02–0.07 Hz	0.50 (0.41, 0.60)	0.52 (0.43, 0.62)	0.669	0.125
0.07–0.20 Hz	1.12 (0.93, 1.30)	1.03 (0.90, 1.16)	0.049*	0.347
0.20–0.50 Hz	1.10 (0.90, 1.30)	1.07 (0.90, 1.24)	0.352	0.119

Data presented are means with 95% confidence intervals throughout the duration of the protocol (∼6 min). MAP, mean arterial pressure; MCAv, middle cerebral artery blood velocity; mmHg, millimeters of mercury; cm/s, centimeters per second; Hz, hertz; a.u., arbitrary units; cm/s/mmHg, centimeters per second per millimeters of mercury. *Different from PRE-BRJ (*P* ≤ 0.05).

#### Cardiovagal baroreflex sensitivity

Blood pressure and cBRS data during Oscillatory-LBNP are presented in Table 2. Systolic, diastolic, and mean arterial pressure during Oscillatory-LBNP were not different between PRE-BRJ and POST-BRJ (all *P* ≥ 0.212). RRI was greater at PRE-BRJ vs. POST-BRJ (*P* = 0.025). Up-cBRS (*P* = 0.600) and Down-cBRS (*P* = 0.511) were not different between PRE-BRJ and POST-BRJ.

**TABLE 2:** Blood pressure and cardiovagal baroreflex sensitivity values during oscillatory lower-body negative pressure before and 3 h after beetroot juice (BRJ) consumption.

Variable	PRE-BRJ	POST-BRJ	*P*-value	Cohen’s d
SBP (mmHg)	128 (122, 134)	125 (118, 133)	0.212	0.283
DBP (mmHg)	74 (69, 78)	73 (68, 77)	0.394	0.133
MAP (mmHg)	94 (89, 98)	92 (87, 96)	0.231	0.250
R-R Intervals (ms)	869 (813, 926)	837 (773, 902)	0.025*	0.316
Up-cBRS	16.5 (12.0, 21.0)	16.0 (11.2, 20.8)	0.601	0.065
Down-cBRS	13.9 (9.9, 18.0)	13.0 (9.8, 16.2)	0.511	0.155

Data presented are means with 95% confidence intervals throughout the duration of the protocol (∼6 min). DBP, diastolic blood pressure; MAP, mean arterial pressure; mmHg, millimeters of mercury; ms, milliseconds; SBP, systolic blood pressure; Up-cBRS, up-up cardiovagal baroreflex sensitivity. *Different from PRE-BRJ (*P* ≤ 0.05).

### Static lower-body negative pressure

#### Hemodynamic response to static LBNP

**FIGURE 4:**
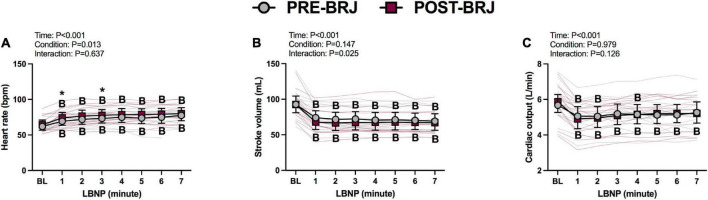
Individual responses and absolute means with 95% confidence intervals for heart rate **(A)**, stroke volume **(B)**, and cardiac output **(C)**, at the last minute of baseline (BL) and during every minute of static lower-body negative pressure (LBNP) before (PRE–BRJ) and 3 h after beetroot juice consumption (POST–BRJ). Linear mixed-effect models were used to compare mean values within a given variable. *Post-hoc* tests using the Bonferroni’s correction for multiple comparisons were used where appropriate. B = different from baseline (*P* ≤ 0.05). *Different from PRE-BRJ (*P* ≤ 0.05).

**FIGURE 5:**
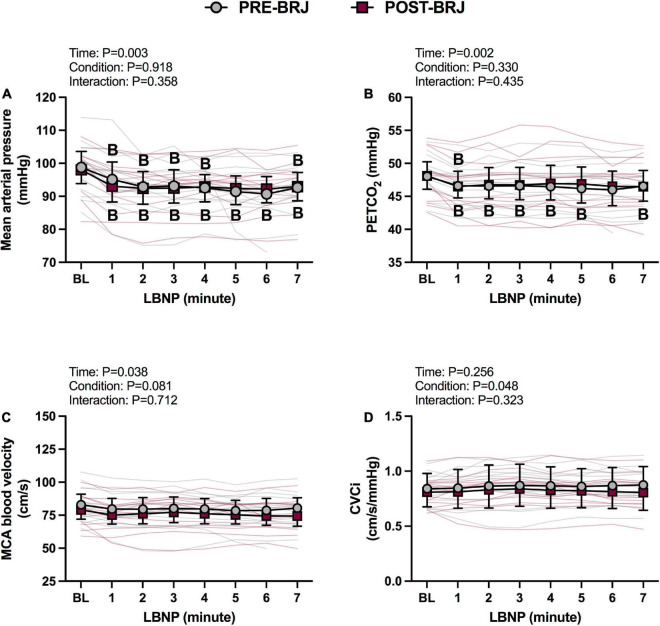
Individual responses and absolute means with 95% confidence intervals for mean arterial pressure **(A)**, end-tidal CO_2_ tension **(B)**, middle cerebral artery blood velocity **(C)**, and cerebrovascular conductance index **(D)** at the last minute of baseline (BL) and during every minute of static lower-body negative pressure (LBNP; –40 mmHg) before (PRE–BRJ) and 3 h after beetroot juice consumption (POST–BRJ). Linear mixed-effect models were used to compare mean values within a given variable. *Post-hoc* tests using the Bonferroni’s correction for multiple comparisons were used where appropriate. B = different from baseline (*P* ≤ 0.05).

Baseline values and values during every minute of Static-LBNP are presented in Figures 4, 5. There were no differences between conditions at Baseline. Heart rate was greater in POST-BRJ vs. PRE-BRJ at minute 1 (*P* = 0.011) and minute 3 (*P* = 0.038) of Static-LBNP (Figure 4A). Stroke volume and cardiac output were not different between PRE-BRJ and POST-BRJ (Figures 4B, C). Mean arterial pressure, and PETCO_2_ were not different between PRE-BRJ and POST-BRJ (Figures 5A, B). Linear mixed-effects models revealed a main effect of time for MCAv, but multiple comparisons did not reveal where specific differences occurred (Figure 5C). Linear mixed-effects models revealed a main effect of condition for CVCi, but multiple comparisons did not reveal where specific differences occurred (Figure 5D).

#### Static cerebral autoregulation

Indices of static CA during Static-LBNP are presented in Table 3. Coherence, phase, and gain were not different between PRE-BRJ and POST-BRJ in the VLF (0.02–0.07 Hz) (all *P* ≥ 0.585), LF (0.07–0.20 Hz) (all *P* ≥ 0.104), or HF band (0.20–0.50 Hz) (all *P* ≥ 0.470). There was an intermediate effect size for phase in the LF band (*d* = 0.660).

**TABLE 3:** Blood pressure, cerebral artery blood velocity, and indices of static cerebral autoregulation during static lower-body negative pressure before and 3 h after beetroot juice (BRJ) consumption.

Variable	PRE-BRJ	POST-BRJ	*P*-value	Cohen’s d
**MAP (mmHg)**	92 (86, 99)	91 (85, 98)	0.642	0.117
**MCAv (cm/s)**	75 (66, 84)	72 (62, 82)	0.123	0.232
**Coherence (a.u.)**				
0.00–0.50 Hz	0.58 (0.51, 0.65)	0.60 (0.50, 0.70)	0.598	0.168
0.02–0.07 Hz	0.49 (0.38, 0.60)	0.47 (0.33, 0.60)	0.689	0.166
0.07–0.20 Hz	0.72 (0.63, 0.82)	0.71 (0.62, 0.81)	0.753	0.088
0.20–0.50 Hz	0.55 (0.44, 0.65)	0.58 (0.45, 0.71)	0.514	0.216
**Phase (degrees)**				
0.00–0.50 Hz	9 (2, 16)	11 (5, 16)	0.568	0.198
0.02–0.07 Hz	55 (35, 76)	61 (45, 77)	0.585	0.235
0.07–0.20 Hz	40 (30, 49)	32 (24, 40)	0.104	0.660
0.20–0.50 Hz	−11 (−24, 2)	−9 (−17, −1)	0.728	0.127
**Gain (cm/s/mmHg)**				
0.00–0.50 Hz	1.02 (0.86, 1.19)	1.06 (0.87, 1.26)	0.574	0.171
0.02–0.07 Hz	0.63 (0.47, 0.78)	0.68 (0.51, 0.85)	0.271	0.249
0.07–0.20 Hz	1.07 (0.85, 1.31)	0.99 (0.82, 1.16)	0.113	0.354
0.20–0.50 Hz	1.08 (0.88, 1.27)	1.16 (0.89, 1.43)	0.470	0.276

Data presented are means with 95% confidence intervals throughout the duration of the protocol (7 min). MAP, mean arterial pressure; MCAv, middle cerebral artery blood velocity; mmHg, millimeters of mercury; cm/s, centimeters per second; Hz, hertz; a.u., arbitrary units; cm/s/mmHg, centimeters per second per millimeters of mercury.

#### Static cardiovagal baroreflex sensitivity

Blood pressure and cBRS data during Static-LBNP are presented in Table 4. Systolic, diastolic, and mean arterial pressure during Static-LBNP were not different between PRE-BRJ vs. POST-BRJ (all *P* ≥ 0.718). RRI (*P* = 0.066), Up-cBRS (*P* = 0.122), and Down-cBRS (*P* = 0.118) during S-LBNP were not different between PRE-BRJ vs. POST-BRJ.

**TABLE 4:** Blood pressure and cardiovagal baroreflex sensitivity during static lower-body negative pressure before and 3 h after beetroot juice (BRJ) consumption.

Variable	PRE-BRJ	POST-BRJ	*P*-value	Cohen’s d
SBP (mmHg)	123 (117, 130)	123 (115, 131)	0.934	0.000
DBP (mmHg)	74 (69, 78)	74 (70, 79)	0.718	0.000
MAP (mmHg)	92 (87, 96)	92 (87, 97)	0.955	0.000
R-R intervals (ms)	800 (724, 875)		768 (682, 853)	0.066 0.252
Up-cBRS	18.0 (13.1, 23.0)	16.0 (10.3, 21.7)	0.122	0.245
Down-cBRS	13.7 (9.9, 17.5)	11.5 (7.9, 15.1)	0.118	0.377

Data presented are means with 95% confidence intervals throughout the duration of the protocol (7 min). DBP, diastolic blood pressure; Down-cBRS, down-down cardiovagal baroreflex sensitivity; MAP, mean arterial pressure; mmHg, millimeters of mercury; ms, milliseconds; SBP, systolic blood pressure; Up-cBRS, up-up cardiovagal baroreflex sensitivity.

## Discussion

We investigated the acute effects of BRJ on CA and cBRS during oscillatory and static LBNP challenges in healthy, young adults. Our primary findings are that 3 h following the consumption of 500 mL of BRJ: (1) indices of dynamic CA are altered during blood pressure oscillations induced at ∼0.02 Hz, but not during a static hypotensive stimulus, and (2) cardiovagal baroreflex sensitivity is not altered during an oscillatory or static hypotensive stimulus.

Beetroot juice did not alter mean arterial pressure during supine rest. Blood pressure has previously been shown to decrease following acute and chronic dietary nitrate supplementation (primarily BRJ), with more pronounced reductions observed in systolic vs. diastolic blood pressure (Webb et al., 2008; Kapil et al., 2010; Vanhatalo et al., 2010; Siervo et al., 2013; Ashor et al., 2017). However, others have reported no change in blood pressure after acute (Lansley et al., 2011; Casey et al., 2015; Curtis et al., 2015; Kroll et al., 2018) and chronic (Blekkenhorst et al., 2018; Oggioni Dgj et al., 2018; Fan et al., 2019) dietary nitrate ingestion (i.e., BRJ, sodium nitrate, and nitrate-rich vegetables). More specifically, BRJ ingestion (140–500 mL) did not alter blood pressure ∼2.5–3 h post-ingestion in healthy adults (Lansley et al., 2011; Casey et al., 2015; Curtis et al., 2015), which is in line with our findings. Our participants exhibited an average change of −2 ± 5 mmHg, and range from −13 mmHg to 8 mmHg. Despite no significant change in blood pressure, however, 500 mL of BRJ does increase circulating nitrates/nitrites within 3 h of consumption (Casey et al., 2015), thus the timing of our measurements were conducted when circulating nitrates/nitrites were likely elevated. Therefore, the observation that BRJ did not lower blood pressure in our study is likely due to studying healthy young adults and not the timing of our LBNP protocols.

We also observed no change in resting MCAv or CVCi from pre- to post-BRJ ingestion. Thus, it appears that BRJ consumption did not change cerebral vascular tone because PETCO_2_ and blood pressure were also not influenced by BRJ. Currently, there is a paucity of investigations in healthy, young adults regarding the acute effects of dietary nitrates on cerebral artery blood velocity and blood flow. A diet high in nitrates increased cerebral perfusion to the frontal lobe, but did not alter global cerebral perfusion in older adults (Presley et al., 2011). Seven-days of dietary nitrate supplementation (BRJ or sodium nitrate) did not alter MCAv in healthy men and women (Fan et al., 2019) or patients diagnosed with a transient ischemic stroke (Fan et al., 2020). Thus, a single bolus of dietary nitrates *via* BRJ does not appear to alter resting peripheral or cerebral hemodynamics in healthy, young adults. More work is needed regarding the dose and frequency of dietary nitrate ingestion on blood pressure and cerebral blood velocity regulation.

### Dynamic cerebral autoregulation during oscillatory LBNP

We assessed dynamic CA using transfer function analysis during blood pressure oscillations induced at ∼0.02 Hz. Although the oscillatory LBNP protocol was not designed to elicit cardiovascular decompensation, all participants tolerated the oscillatory LBNP protocol before and after BRJ. Throughout the six cycles of oscillatory LBNP, we did not observe differences in the mean arterial pressure, PETCO_2_, and MCAv responses between PRE- and POST-BRJ (Figures 3A–C). However, there was a condition effect for MCAv (*P* = 0.044), as MCAv was lower throughout oscillatory LBNP. Despite no statistical differences in the mean arterial pressure and MCAv responses to oscillatory LBNP, there were differences in some transfer function analysis indices of dynamic CA. In the VLF band, coherence was greater following BRJ (Table 1), which suggests a stronger relation between blood pressure and MCAv fluctuations. Despite a stronger relation, VLF phase and gain did not change after BRJ ingestion (Table 1). Thus, the cerebral arterioles buffered VLF oscillations in blood pressure similarly pre- and post-BRJ. We did find that LF gain decreased by ∼0.09 cm/s/mmHg after BRJ, but phase and coherence were not altered. A decrease in LF gain has been reported following 7-days of sodium nitrate supplementation in men, but not women, at rest (Fan et al., 2019). Although we did not examine sex differences, we controlled for menstrual cycle to avoid the potential confounding factor of estrogen on endothelial NOS activity (McNeill et al., 2002), whereas the other investigators did not as their intervention was conducted over 7 days. It is possible that the decrease in LF gain following dietary nitrate ingestion is an improvement in CA. But, it must be considered that the LF band (0.07–0.20 Hz) does not align with our blood pressure challenge (∼0.02 Hz) so these data must be interpreted cautiously. Future work should aim to determine if acute BRJ consumption alters CA during a LF dynamic blood pressure challenge (e.g., O-LBNP or squat-stand maneuvers performed at 0.10 Hz).

### Static cerebral autoregulation during constant LBNP

We also assessed CA during a constant pressure perturbation to lend insight on the static control of cerebral blood flow and blood pressure. Static LBNP decreased mean arterial pressure by ∼6 mmHg (range: 0 to −40 mmHg) at PRE-BRJ and ∼5 mmHg (range: 0 to −13 mmHg) at POST-BRJ. The wide range at pre-BRJ was contributed by one male participant that exhibited large reductions in blood pressure at minute 5 and 6 at −40 mmHg before LBNP was terminated due to experiencing relative bradycardia associated with a decreasing blood pressure. Interestingly, this participant tolerated static LBNP following BRJ. Regardless, mean arterial pressure throughout the 7 min of LBNP was not different between pre and post-BRJ (Figure 5A). We observed a similar response in PETCO_2_, as BRJ did not alter PETCO_2_ during LBNP compared to pre-ingestion. Utilizing an FiCO_2_ of 0.03 attenuated potential reductions in PETCO_2_ during LBNP, as PETCO_2_ was ∼1–2 mmHg lower than baseline during every minute of LBNP (Figure 5B). More pronounced reductions in PETCO_2_ may have occurred during LBNP if we did not add CO_2_ to the inspirate. We previously observed ∼5 mmHg decrease in PETCO_2_ during 5 min of LBNP in healthy men and women (Worley et al., 2021). Despite reductions in mean arterial pressure and PETCO_2_, MCAv was maintained throughout LBNP at pre- and post-BRJ ingestion (Figure 5C). These mean values suggest that cerebral autoregulatory capacity was not altered by BRJ ingestion. Indices of static CA *via* transfer function analysis support the results of the analyses on the mean data as coherence, phase, and gain did not differ in any frequency band from PRE- to POST-BRJ (Table 3). Evidence is scant regarding the acute effects of dietary nitrate on CA during a sustained and acute hypotensive stimulus. Consuming 500 mL of BRJ (∼750 mg of nitrate) has been shown to reduce the cerebral augmentation index during aerobic exercise performed at 40% and 80% of peak oxygen consumption as well as lower SBP at rest 2 h following ingestion 66 (Curry et al., 2016). It is difficult to compare our results to this investigation as aerobic exercise poses a different challenge to cerebral vascular resistance and blood flow compared to LBNP. Our data align more with Horiuchi et al. (2022) who had participants ingest 140 mL of BRJ per day for 4 days. CA in the internal carotid artery (ICA) was not altered using the thigh-cuff deflation technique that induces a rapid decrease in blood pressure. Although we also observed no change in CA, the vessel of interest (ICA vs. MCA), analysis technique (rate of regulation vs. transfer function analysis), mode of test (thigh cuff deflation vs. O-LBNP), and BRJ dose (4 days of supplementation vs. acute and volume) were different. Nevertheless, our healthy participants maintained CA function following the acute ingestion of BRJ.

### Dynamic and static cardiovagal baroreflex sensitivity

Utilizing the sequence method, we hypothesized that cBRS would decrease (i.e., worsen) following BRJ ingestion in healthy, young adults. Contrary to our hypothesis, we observed no change in Up-Up or Down-Down cBRS after BRJ during static or oscillatory LBNP. During oscillatory LBNP, we observed no change in blood pressure between conditions, but RRIs decreased from pre to post-BRJ (Table 2). Although RRIs during the static LBNP protocols were not statistically different between conditions, the RRI responses (*P* = 0.07) were similar to the oscillatory LBNP responses. Yet, blood pressure and cBRS during the static LBNP protocols were not different between conditions (Table 4). Although we did not observe a change in cardiovagal baroreflex sensitivity following BRJ consumption, the acute administration of a nitric oxide donor did increase baroreceptor sensitivity in rabbits (Matsuda et al., 1995), whereas sublingual nitroglycerin reduced spontaneous cBRS immediately following administration (∼10 min) in healthy participants (Hamaoka et al., 2021) and 6-days after administration (Gori et al., 2002). Our findings likely differ from others due to the inorganic nitrate administered, time of testing post-administration, and how baroreflex sensitivity was measured (e.g., spontaneous vs. perturbation).

### Experimental considerations

There are several methodological considerations that should be taken into account when interpreting our data. First, we removed the transcranial Doppler ultrasound probe between testing sessions. This may alter the signal between testing time points. However, we utilized standard insonation techniques set forth by Willie et al. (2011) and recorded the depth, gain, and location (with indelible ink) to ensure the insonation angle was closely matched. Second, we did not establish baseline dynamic CA while participants were breathing room air. Lower body negative pressure increases ventilation (Koehle et al., 2010) and decreases end-tidal CO_2_ content (Ahn et al., 1989; Bronzwaer et al., 2017; Rosenberg et al., 2021), which can exacerbate reductions in intracranial artery blood velocity. Therefore, we bled in a mild hypercapnic gas (3% CO_2_, 21% O_2_, balanced with N_2_) to the inspirate in an attempt to attenuate reductions in PETCO_2_ during LBNP protocols. The mild hypercapnic gas was inhaled for 5 min of supine baseline to allow for a new stable PETCO_2_ and baseline to be achieved prior to LBNP initiation. It has previously been reported that hypercapnia impairs dynamic CA, but this has been shown when PETCO_2_ is 5 mmHg greater than resting baseline (Aaslid et al., 1989; Zhang et al., 1998; Ainslie et al., 2005). On average, 3% CO_2_ increased PETCO_2_ by ∼3 mmHg (PRE-BRJ) and ∼4 mmHg (POST-BRJ) from room air supine rest. We speculate that CA was not impaired due to the modest increases in PETCO_2_ that likely did not alter MCA diameter (Verbree et al., 2014; Kellawan et al., 2016), but we cannot confirm this without air breathing conditions. Nevertheless, PETCO_2_ did not differ during supine rest, oscillatory LBNP, or static LBNP before or after BRJ consumption. Second, we did not have a placebo condition to more clearly determine the effects of BRJ ingestion on CA that may be influenced by time alone (i.e., tested 3 h apart). Daily fluctuations in CA are poorly understood, but it appears that spontaneous autoregulation remains stable during the daytime hours (8 am–8 pm) in healthy individuals (Guo et al., 2018). All of our experimental visits took place within this time frame. Third, we did not measure plasma nitrate or nitrite to determine the magnitude of increase after consuming 500 mL of BRJ. Using the same dose of a commercially available BRJ (500 mL of Biotta), Casey et al. (2015) reported a threefold increase in plasma nitrite 3 h following ingestion in a group of participants with similar demographics as our study. Thus, it is very likely that circulating nitrites were elevated in our study. Fourth, we did not randomize the LBNP protocols. There is a possibility that prolonged inhalation of hypercapnic gas may have influenced LBNP protocols differently. However, it was not our objective to compare CA or cBRS responses between LBNP protocols. Finally, females were tested during the early follicular phase to control for the influence of sex hormones on endothelial NOS activity (Miller and Duckles, 2008; Duckles and Miller, 2010). It is currently unclear if there is an interaction between menstrual cycle, dietary nitrates, and CA. Sex differences have been observed in CA, but this does not appear to be affected by menstrual cycle phase (Favre and Serrador, 2019). However, Fan et al. (2019) had participants consume sodium nitrate (10 mg/kg/day) for 7 days and observed a similar response in LF mean gain (no change) and LF phase (increased) between men and women. However, others have observed no sex differences in CA at rest and following 7 days of dietary nitrate supplementation (Fan et al., 2019). Fan et al. (2019) observed similar autoregulatory responses between men and women following dietary nitrate supplementation (sodium nitrate; 10 mg/kg/day for 7 days), with both sexes exhibiting contradictory alterations in CA (i.e., decrease in gain and phase).

## Conclusion

In summary, during an oscillatory blood pressure challenge, we observed an increase in VLF coherence, which may indicate a stronger linear relation between blood pressure and middle MCAv following BRJ ingestion. However, transfer gain and phase were not altered within this frequency band, indicating that there was no change in cerebral autoregulatory capacity. After BRJ, we did observe a slight reduction in LF gain during Oscillatory-LBNP. It is unclear if this is indicative of improved dynamic CA since the blood pressure oscillations were induced at a very low frequency. Indices of CA did not change during constant LBNP which indicates that static CA is not acutely altered with BRJ. Lastly, BRJ did not alter cBRS during dynamic and static LBNP in healthy, young adults. Therefore, a single 500 mL bolus of BRJ does not appear to acutely improve dynamic or static CA or cBRS during LBNP in healthy, young adults.

## Data availability statement

The raw data supporting the conclusions of this article will be made available by the authors, without undue reservation.

## Ethics statement

The studies involving human participants were reviewed and approved by the Institutional Review Board at the University at Buffalo. The patients/participants provided their written informed consent to participate in this study.

## Author contributions

All authors listed have made a substantial, direct, and intellectual contribution to the work, and approved it for publication.
